# Carbohydrate Nutrition and the Risk of Cancer

**DOI:** 10.1007/s13668-019-0264-3

**Published:** 2019-03-20

**Authors:** Christian A. Maino Vieytes, Hania M. Taha, Amirah A. Burton-Obanla, Katherine G. Douglas, Anna E. Arthur

**Affiliations:** 1 Division of Nutritional Sciences, University of Illinois at Urbana-Champaign, 905 S Goodwin Ave, 386 Bevier Hall, Urbana, IL 61801 USA; 2 Department of Food Science and Human Nutrition, University of Illinois at Urbana-Champaig, Urbana, IL USA; 3 Carle Cancer Center, Carle Foundation Hospital, Urbana, IL USA

**Keywords:** Diet, Cancer survival, Cancer prevention, Epidemiological studies, Fiber, Glycemic index

## Abstract

**Purpose of Review:**

This review summarizes a selection of epidemiologic research assessing the associations between carbohydrate intake and cancer incidence and survival. Evidence for plausible biological mechanisms is also considered.

**Recent Findings:**

The mechanistic paradigm explaining the relationship between carbohydrates and cancer risk has been contested by numerous observational studies.

**Summary:**

Carbohydrates have conventionally been ascribed a deleterious role in the field of cancer research due to previous preclinical findings. A breadth of studies suggests that complex carbohydrate intake is inversely associated with risk of a number of cancer types. Data from studies assessing simple carbohydrates and cancer risk are mixed. Furthermore, recommendations for subsequent studies are framed.

## Introduction

An estimated 606,880 deaths in the USA will be attributable to cancer in 2019 [1]. Cancer is projected to surpass infectious and other non-communicable chronic diseases as the leading cause of death in every country during the twenty-first century [2]. Cancer incidence has plateaued and survival rates have increased in the USA, primarily due to advances in detection and treatment [1]. However, burgeoning global incidence rates and a rapidly growing population of cancer survivors highlight the severity of the public health and economic challenges posed by this disease [2]. Public health approaches that are low-cost and prioritize cancer prevention through lifestyle and behavioral modifications are urgently needed [3••]. Diet is one modifiable lifestyle factor that has garnered attention due to substantial and growing evidence of its ability to influence cancer risk.

**Fig. 1:**
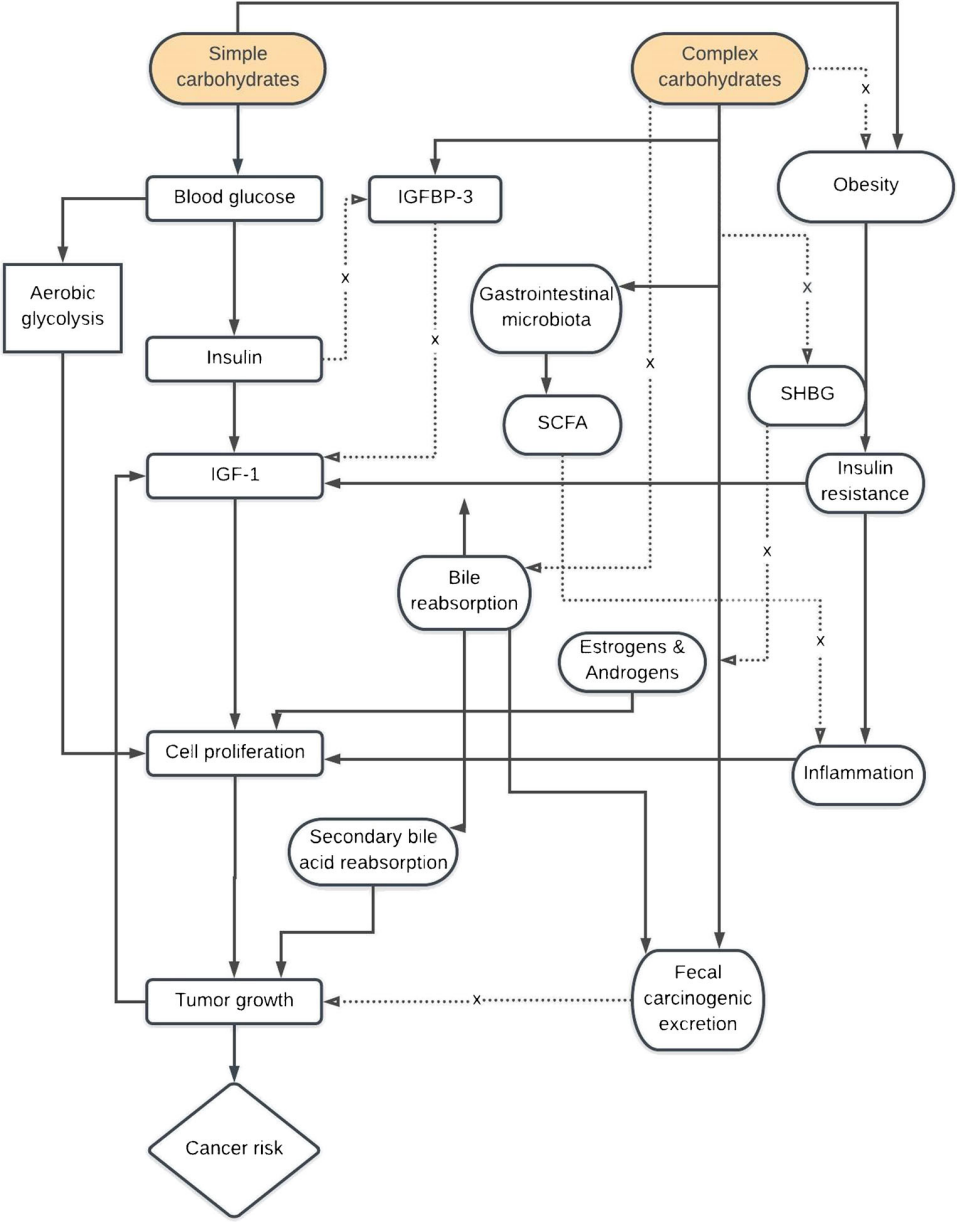
Posited mechanistic frameworks implicating dietary carbohydrates and cancer risk. Simple and complex carbohydrates have different mechanisms that induce variable signaling pathways, which may affect cancer risk. Simple carbohydrates may increase risk by activating the insulin-IGF-1 axis and by employing aerobic glycolysis as the primary energy-harvesting pathway (known as the Warburg effect). On the contrary, complex carbohydrates may reduce the risk by disrupting the insulin/IGF-1 axis, quenching bioavailable androgenic and estrogenic factors, increasing fecal excretion of carcinogens, and modulating the gastrointestinal microbiota. Black arrows indicate the stimulation of a pathway, dotted lines indicate the inhibition of a pathway, and broken lines indicate a negative feedback

Carbohydrate intake is one aspect of diet that has been hypothesized to modulate cancer risk depending on the amount and type consumed. Carbohydrates are a broad category of biomolecules, which, in their monosaccharide forms, function as preferred cellular energy substrates [4]. Aside from their crude function, carbohydrates exert a comprehensive set of effects at the cellular, physiological, and ecological levels. Remarkable among these are microbial and epigenetic modulations as well as endocrine and systemic alterations resulting from their consumption that may potentially influence cancer risk and progression [4, 5]. Despite in vitro and animal research providing evidence of mechanisms through which carbohydrates may impact cancer risk, the epidemiologic evidence linking dietary carbohydrates to cancer development and progression has remained unclear. In this review, we present an overview of the mechanistic frameworks through which carbohydrates are hypothesized to exert their influence on cancer risk (Fig. 1). We then summarize recent epidemiologic evidence linking dietary carbohydrates, mainly simple and complex carbohydrates, with primary and tertiary prevention parameters for a variety of primarily adiposity-related cancer types. Finally, we provide our conclusions and suggested directions for future research that can ultimately inform public health and medical recommendations regarding carbohydrate consumption and cancer risk (Table 1).

**Table 1:** Review highlights

• Existing mechanistic paradigms describing the relationships between dietary carbohydrates and cancer are biologically plausible but need corroborating evidence in humans before translation to public health recommendations and clinical guidelines. • The majority of observational data support a beneficial role for complex carbohydrates and fiber, especially from whole-grain sources, in the primary prevention of a number of cancers. • Associations ascertained from observational data for the consumption of simple carbohydrates and cancer risk have been mixed. • Inconsistencies in study design and methodologies are problematic when drawing conclusions and conducting meta-analyses across studies. • Data relating carbohydrate intake and tertiary prevention of cancer, specifically recurrence and mortality, have been limited and should be a primary focus of subsequent observational and experimental studies. • There have been limited dietary intervention trials assessing the impact of carbohydrate on cancer-related biomarkers. In the short-term, RCTs should be conducted to determine how carbohydrate intake affects biomarkers of different cancer types (e.g., SHBG in PCa). • More prospective studies and RCTs are needed to clarify the long-term relationship between carbohydrate intake and cancer progression and prognosis.	

## Mechanistic Frameworks

Early findings from experimental in vitro and in vivo models enhanced understanding of the acute physiological effects of carbohydrates. On the basis of these discoveries, a series of etiological frameworks tying their consumption to cancer risk were hypothesized. Owing to the work of Otto Warburg, a deleterious role, albeit a simplistic one, was ascribed to dietary carbohydrates; a hypothesis that, as we shall examine, has seemingly been only moderately supported by observational data. A more coherent framework, primarily implicating insulin-signaling networks, was born out of subsequent research. These hypotheses, detailed below, have provided a foundation that has motivated a vast collection of the observational studies we address in this review.

### The Warburg Effect and Glucose Metabolism

Exploitation of glycolytic pathways and their corresponding machinery are appreciable metabolic adaptations that cancer cells use to enhance and perpetuate their survival and proliferative capabilities. Specifically, tumor cells transition from aerobic cellular respiration to aerobic glycolysis as the primary energy-harvesting mechanism [6]. This highlights a paradoxical phenomenon whereby the energy demands of the tumor are met through a shift to a largely inefficient means of energy production: that of high rates of glucose fermentation [6]. This phenomenon was identified by Nobel Laureate Otto Warburg in 1924 in his renowned “Warburg Hypothesis” and serves as a framework that has burgeoned through the work of subsequent scholars since its inception. Despite the relative disadvantages of the glycolytic system compared to oxidative phosphorylation, this adaptation confers various advantages to cancer cells, namely, precursor production, enhanced function in hypoxic environments, promotion of serine/threonine kinase (Akt) activity, promotion of the K-Ras oncogene that upregulates GLUT1, and modification of the acidic tumor microenvironment [7–11]. Nevertheless, the contributions of Warburg and his colleagues to the field of metabolic adaptation lend themselves to the realm of nutrition and cancer and have generated hypotheses regarding the utility of dietary carbohydrates in cancer treatment protocols [12, 13].

### Insulin and the IGF-1 Axis

The insulin/IGF-1 signaling axis plays a critical role in glucose metabolism and subsequently modifies cellular proliferation and growth [14]. Insulin, through its interaction with isoforms of insulin receptor (IR), is central to glucose uptake and energy homeostasis. Experimental models have demonstrated that insulin-IR signaling activates signal transduction pathways, including mTOR and PI3k/Akt, directly associated with cellular proliferation [14]. In particular, overexpression of IR and its interaction with circulating insulin is a hallmark of many cancers [15, 16]. Likewise, observational studies have established a clear link between hyperinsulinemia and increased risk of adiposity-related cancers [17–19].

Many anabolic functions of the IGF-1 axis, such as the activation of PI3K/Akt, parallel those of the insulin-IR signaling cascade [14, 20]. Dysregulation of this signaling pathway has been implicated in a variety of cancers. Critically, the availability of free and bioactive IGF-1 is modified, primarily, by concentrations of IGF-1 binding proteins (IGFBP) [14]. Evidence of associations between IGFBP and IGF-1 levels and cancer phenotypes at the population level have been reported in the literature [21–25]. The links between dietary carbohydrates and cancer risk are hypothesized to involve mechanisms that directly implicate players in insulin-mediated pathways across various tissue types as well as through modulation of IGF-1 bioactivity [26–28].

## Primary Prevention of Cancer: Simple Carbohydrates

Consumption of simple sugars, glycemic index (GI) and glycemic load (GL), has been hypothesized to increase cancer risk, although findings have been mixed. Makarem et al. analysis of the Framingham cohort reported no significant associations between GI or total carbohydrates and risk of several adiposity-related cancers [29]. Nonetheless, the following subsections are dedicated to reviewing current research examining simple carbohydrates and primary risk of various cancer types.

### Colorectal Cancer

An ecological analysis undertaken by Grasburger and his colleagues on observational data collected across 39 European countries reported weak and moderate positive associations between refined sugar consumption and CRC incidence in men and women, respectively [30]. Giovannucci hypothesized that the synergistic combination of insulin resistance and consumption of high GL foods was responsible for cuing hyperinsulinemia, which can amplify growth factors and mitogenic response [31]. Giovannucci’s hypothesis also relied on a number of other concomitant criteria, mainly that of increased fasting plasma glucose, central obesity, and a paucity of fiber-rich foods being consumed [31]. Epidemiologic studies have, nonetheless, focused on discerning the relationship between simple sugars and CRC.

Using GL as a predictor, Zelenskiy et al. case-control study reported significantly increased odds of CRC in the second through fourth upper quartiles of GL consumption [32]. The effect was particularly pronounced in the elderly stratum, insinuating effect modification by age. The relationships between GI, GL, and colorectal cancer risk were broached in longitudinal designs as well. Higginbotham et al. prospective analysis of the Women’s Health Study cohort revealed significant positive associations between GI, GL, total carbohydrate, non-fiber carbohydrate, sucrose, fructose, and CRC risk [33]. An analysis of the European Prospective Investigation into Cancer and Nutrition (EPIC) Italy cohort suggested a 35% increase in CRC risk associated with increasing quartiles of dietary GI. High-GI carbohydrate, another independent predictor, was associated with a 45% increased risk of CRC [34]. Stratification by tumor site revealed variable spatial effects according to the simple carbohydrate predictor used. High-GI carbohydrate was associated with an increased risk of tumor development in the proximal colon but not the distal or rectal regions. Similarly, although not a significant main effect, higher GL consumption was associated with a two-fold increase of proximal colon tumor risk.

Arguably, the most comprehensive set of meta-analyses conducted to date, by Reynolds et al., included 185 studies that examined several carbohydrate predictors (i.e., dietary fiber, whole-grains or pulses, dietary GI, or GL) and their associations with a range of clinical outcomes, including incidence of many adiposity-related cancers (CRC, breast, endometrial, esophageal, and prostate cancer) and total cancer mortality. Both randomized controlled trials (RCTs) and prospective cohort studies were considered. Results from the meta-analysis on GI and CRC incidence, which included 10 studies, reported a non-significant but 5% increased risk associated with greater consumption of higher GI foods [35]. The only study included in their analysis that examined GI in relation to total cancer mortality also reported null findings.

### Prostate Cancer

Studies examining the relationship between high consumption of simple carbohydrate and the risk of PCa have been inconsistent. While some studies suggested a positive association between simple carbohydrate consumption and risk of PCa, others reported null associations. The two longitudinal studies cited by Makarem et al. in their systematic review come from Giovannucci et al. and Drake et al. who reported on the Health Professionals Follow-Up and Malmö Diet and Cancer cohorts, respectively. Notably, Giovannucci et al. found a significantly reduced risk of advanced PCa associated with higher fructose consumption [36]. In contrast, Drake et al. reported that the lowest consumption of monosaccharides corresponded with a 31% decreased risk of symptomatic prostate cancer (i.e., exhibiting lower urinary tract or other malignancy-related symptoms) [37]. Another meta-analysis by Zhai and colleagues found no significant associations between total carbohydrate intake and PCa risk, even when stratifying by study design (case-control and cohort) [38]. These findings were further substantiated by Fan et al. who failed to report significant associations between dietary carbohydrate consumption and risk of advanced and non-advanced PCa on data from 22 studies that included 6 prospective cohort and 16 case-control designs [39•].

### Breast and Other Female Reproductive Cancers

The data on the risk of female reproductive cancers in relation to simple carbohydrate intake, primarily GL and GI, have been inconsistent. The ecological associations reported by Grasburger et al. indicated moderate positive linear relationships between the consumption of refined sugar and breast cancer [30]. An earlier meta-analysis conducted by Barclay et al. on 37 prospective cohort studies, 7 of which examined breast cancer as an outcome, assessed the relationships between GL, GI, and chronic disease risk. Results showed an 8% increase in breast cancer risk for high-GI and a 40% increased risk of endometrial cancer with high GL, based on three studies [40]. One study examining ovarian cancer risk reported a significant positive association with GL, but not with GI [41]. Several data collection and conclusion errors in that study were identified [42]. A subsequent, updated meta-analysis pooled data from six prospective cohort studies examining associations between GI and GL with breast cancer risk. The results suggested null associations with GI and GL in both premenopausal and postmenopausal females [43]. An underlying limitation among these studies was their lack of stratification by estrogen receptor (ER) status in analyses.

Mullie et al. were the first to conduct a meta-analysis that adjusted for ER status. However, they found that ER status did not change relative risk estimates and reported modest increases in risk between the highest versus lowest levels of GI and GL regardless of ER status [44]. In contrast, Schlesinger et al. most recent meta-analysis demonstrated weak, positive associations between breast cancer risk and GI and GL [45]. Evidence of effect modification by ER receptor status was observed, where there was an 11% increased risk with each 50-g/day increase in total carbohydrate for ER− participants but not for ER+.

Makarem et al. 2018 systematic review identified 11 prospective cohort studies that examined the following carbohydrate variables in relation to risk of breast, endometrial, and ovarian cancers: total sugar, added sugar, fructose, sucrose, sugary foods, and sugar-sweetened beverages [46••]. The study findings were inconsistent, with several reporting significantly increased risks while others reported null findings [46••, 47–49].

## Primary Prevention of Cancer: Dietary Fiber and Other Complex Carbohydrates

### Colorectal Cancer

Citing evidence of disparate CRC incidence among rural African and Western populations, Denis Burkitt postulated fiber intake as responsible for these observations given the ubiquitous and significantly higher consumptions exhibited by the former group [50]. Since then, several longitudinal studies have corroborated his postulate, and ample experimental studies have suggested potential mechanisms [51]. Early findings implicating fecal weight and intestinal transit time as crucial predictors of colon cancer risk suggested a critical role for insoluble fiber and demonstrated the role of lignin as a nitrite-scavenger [50, 52, 53]. Decreased bile reabsorption, enhanced fecal excretion of carcinogens, and promotion of the gastrointestinal microbiota, specifically the production of short chain fatty-acid (SCFA)-producing species and greater ecological diversity, are also generally accepted as proposed mechanisms imparting benefit [54•, 55–57]. O’Keefe summarizes the immunomodulatory and anti-proliferative effects of enteric metabolites of complex carbohydrates, including the range of SCFA [54•].

Initial epidemiologic research examining fiber and primary prevention of CRC risk was mixed. A meta-analysis of 13 prospective cohort studies by Park et al. was unable to ascertain a significant inverse relationship between fiber intake and CRC risk [58]. An analysis of the multiethnic cohort study determined a significant inverse association for fiber, but in stratified analyses, this was significant only for men [59]. Park et al. addressed the sex-fiber interaction in a subsequent study within the same cohort, demonstrating that the relationship between dietary fiber and CRC risk in women was confounded by menopausal hormone therapy (MHT). MHT use was identified as independently being associated with a 19% reduction in CRC risk. MHT-ever users who consumed high dietary fiber had an even lower, dose-dependent risk [60]. A commensurate significant association was not observed in MHT-never users.

Evidence of a protective association with high fiber intake emerged from an analysis by Murphy et al. of EPIC data. Specifically, every 10-g increase in total fiber consumption was associated with a 13% reduction of risk [61]. In a meta-analysis of 11 European and American prospective cohort studies examining the associations of fiber with subsite-specific CRCs, Ma et al. demonstrated significant risk reduction with high intake for proximal and distal CRC tumors [62••]. In a cross-sectional sample of individuals undergoing colonoscopy screening, Shaw et al. demonstrated a significant inverse association between total fiber and risk of adenomatous polyps [63]. Associations did not differ when assessing soluble and insoluble fiber separately. Similarly, based on data pooled from 22 longitudinal studies, Reynolds et al. reported a significant inverse association between total fiber and CRC, where an 8% reduction in CRC incidence was observed with every additional 8 g of fiber consumed [35].

### Prostate Cancer

PCa progression and risk have been previously correlated to the IGF-1 system. Its associations with increased concentrations of IGFBP and IGF-1 were demonstrated through several case-control studies [64–66]. A study from the Seventh Day Adventist Cohort demonstrated a significantly greater steroid-hormone binding capacity and fecal excretion in the vegan group that was consuming the greatest quantities of dietary fiber and, specifically, the insoluble lignin fiber type [67]. This drew support for the hypothesis that PCa risk could be mitigated through androgen modulation by dietary fiber. Clinical studies have shown an effect of dietary fiber on testosterone levels [68]. Tymchuk et al. low fat/high fiber/high complex carbohydrate dietary intervention showed an inverse correlation between levels of insulin and sex-hormone binding globulin (SHBG) in pre-intervention samples [69]. Post-intervention samples demonstrated higher SHBG levels and lower levels of insulin, suggesting a potential reduction in PCa risk. The study also comprised an exercise intervention and an overall reduction in animal protein consumption, which may have confounded results.

More recent meta-analyses on epidemiologic data suggested a null association between fiber intake and PCa risk. Sheng et al. meta-analysis of 17 observational studies reported a significant protective association between fiber and PCa risk when considering only case-control studies [70]. When examining cohort studies alone, or combined with case-control data, the association was null. Wang et al. meta-analysis on 27 cohort and case-control studies also showed no significant relationships between dietary fiber, whole-grains, carbohydrate, GI, or GL and PCa risk [71]. Subgroup analyses by fiber type were null. In fact, an unexpected significant positive association between whole-grain consumption and PCa risk was reported for cohort data.

### Head and Neck Cancers

A pooled analysis of seven individual case-control studies participating in the International Head and Neck Cancer Epidemiology Consortium (INHANCE) characterized dietary patterns and assessed their associations with oropharyngeal and laryngeal cancers. Results of their analysis revealed that an “antioxidants and fiber” pattern was associated with a significant inverse association with these cancer types [72]. In a similar dietary pattern analysis examining cases and controls of men with oral, pharyngeal, or laryngeal cancers in Uruguay, Deneo-Pellegrini demonstrated increased odds for those ranking high on the “meat based” and “starchy” patterns [73]. Interestingly, the former factor was characterized by high loadings of starch and dietary fiber, which would call into question a protective effect of these nutrients. However, the factor loading matrix for the “starchy pattern” revealed considerable loading by white bread and refined sources, hinting at potential confounding. The researchers acknowledged the inconsistency and called for further investigation. Kawakita et al. reported an inverse relationship between fiber intake and oropharyngeal and laryngeal cancers in ten pooled case-control INHANCE studies [74]. Despite the results, considerable heterogeneity among study designs was a limitation.

The most comprehensive longitudinal cohort study of complex carbohydrates and head and neck cancer risk to date comes from Lam et al., whose analysis on the NIH-AARP Diet and Health Study cohort revealed a significant inverse association between fiber and whole grains with head and neck cancer risk over the 11-year follow-up period [75]. Results did not change when stratifying by fiber and grain types, but effect modification by sex was observed, where there was no significant association in men.

### Breast and Other Female Reproductive Cancers

The systematic review and meta-analysis by Chen et al. included 24 individual observational studies and reported a significant inverse association between fiber intake and breast cancer risk [76•]. Notably, the majority of studies were longitudinal (*n* = 20) and included both pre- and post-menopausal cohorts.

## Dietary Carbohydrates and Tertiary Prevention

The scope of evidence linking carbohydrate intake to cancer recurrence and survival is limited. However, a selection of studies addressing survival outcomes is summarized below.

### Colorectal Cancer

An analysis of the EPIC cohort that assessed pre-diagnosis associations between dietary fiber and survival of non-metastatic CRC cases reported null results [77]. However, in the Reynolds et al. set of meta-analyses, high whole-grain intake was associated with a 16% reduction in overall cancer mortality, based on data from seven longitudinal studies, and a 13% reduction in CRC-specific mortality, based on five longitudinal studies [35]. Similarly, total fiber was associated with a 13% reduction in cancer mortality, based on five longitudinal studies.

Song et al. demonstrated dose-response patterns and significant reductions in mortality risk with high fiber intake in CRC survivors pooled from the Nurses’ Health Study and the Health Professionals Follow-up Study cohorts. Fully adjusted post-diagnosis models suggested a 22% and 14% reduced risk of CRC-specific and all-cause mortality, respectively, for each 5-g increase in dietary fiber [78••]. With every 5-g increase in daily cereal fiber consumption from pre- to post-diagnosis, an 18% reduction in CRC-specific mortality risk was observed. In subanalyses of post-diagnosis intake, cereal fiber was inversely associated with CRC-specific and all-cause mortality while vegetable fiber was inversely associated with all-cause mortality only. Higher post-diagnosis whole-grain consumption was also associated with reduced risk of CRC-specific and all-cause mortality. In sum, their findings are consistent with the general trend of inverse associations between total fiber and cereal fiber reported in the primary prevention literature.

### Head and Neck Cancers

A prospective cohort study of head and neck cancer patients by Arthur et al. examined associations between pre- and post-treatment carbohydrate intake and recurrence, disease-specific, and all-cause mortality. Results indicated that high pre-treatment intake of total carbohydrate, total sugar, glycemic load, and simple carbohydrate foods (i.e., refined grains, desserts, and sugar-sweetened beverages combined) were significantly associated with increased risk of all-cause mortality [79••]. Total carbohydrates and total sugar were inversely associated with disease-specific mortality. Post-treatment analyses revealed null associations. Starchy foods (i.e., grains, potatoes, legumes, and other vegetables combined) were inversely associated with risk of recurrence, disease-specific, and all-cause mortality.

### Breast and Other Female Reproductive Cancers

A nested case-control study conducted by Emond et al. investigated the association between carbohydrate intake and breast cancer recurrence [80]. Cases were dichotomized into IGF-1 receptor positive (+) or negative (−) tumors. The analysis revealed that IGF-1(+) status independently predicted a greater likelihood of a recurrence event [80]. Increased carbohydrate intake also independently predicted the same outcome. However, the stratified subanalysis showed that carbohydrate intake was positively associated with recurrence in IGF-1(+) participants, while the same associations were null for IGF-1(−) participants. This suggests a potentially beneficial role for personalized carbohydrate recommendations on the basis of tumor molecular characteristics. RCTs are needed to substantiate this.

A longitudinal study in ovarian cancer survivors suggested an inverse association between complex carbohydrate consumption and mortality [81]. Playdon et al. reported inverse associations between pre-diagnosis fiber intake and GI with mortality in the Australian Ovarian Cancer Study cohort [81]. Fecal excretion of circulating endocrine factors, which have been implicated in the progression of female-reproductive tumors, may potentially constitute the mechanistic scheme underlying this relationship. In a RCT of breast cancer survivors by Rock et al., fiber intake was independently associated with a reduction of total and bioavailable estradiol levels [82]. This biologically plausible hypothesis warrants further consideration in subsequent studies.

## Conclusion

The majority of the research on carbohydrates and cancer risk reviewed herein was derived largely from case-control and cohort designs, which makes it impossible to draw definitive conclusions of causality. Moreover, the breadth and strength of the evidence were most convincing for associations between fiber and complex carbohydrate consumption with CRC risk, although a similar association is suggested in other cancer types. Consistent with the findings of this review, the World Cancer Research Fund/American Institute for Cancer Research (WCRF/AICR) now recognizes dietary fiber as a probable protective agent against colorectal carcinogenesis [3••]. The data on simple carbohydrates, overall, was mixed for all cancer types examined. The data on carbohydrate consumption on survival outcomes in cancer populations is limited.

There is an urgent need for additional prospective cohort studies and RCTs, particularly for cancer types other than CRC and of tertiary cancer prevention. There was significant heterogeneity across the studies reviewed and thus adopting consistent methodologies for studying the associations between carbohydrate intake and cancer risk are needed. Doing so will lead to more consistent results that can be used to design rigorous RCTs that will ultimately inform public health and clinical recommendations. Specifically, consistent examination of various subtypes of carbohydrates should be considered in future research. Many studies considered carbohydrates as either an all-encompassing predictor or in stratified classifications (i.e., whole grains, fiber, or complex carbohydrates), albeit at times confounded by inappropriate groupings (e.g., white bread). Moreover, it must be noted that obesity is an accepted etiologic factor implicated in several of the cancer types considered in this review [3••]. As such, it is imperative to contemplate that other lifestyle and dietary variables are justifiably important when interpreting results and drawing conclusions.

Clinical recommendations based off this review need be tempered. The data do not support any conclusion regarding the feasibility or efficacy of extreme carbohydrate-restricting dietary protocols, such as the ketogenic diet, despite the fact that several experimental mouse models have highlighted its benefits when implemented concomitantly with pharmacological treatments. Several recent reviews on the topic of the ketogenic diet and cancer have been published, and thus, it was not considered in the current review [13, 83, 84]. Another consideration follows from the recent prospective cohort analysis by Seidelman et al. that found a significantly increased risk of all-cause mortality associated with both very high and very low carbohydrate intakes, suggesting that perhaps not only the type, but also the amount of carbohydrates consumed is important for health [85••].

In sum, the body of evidence related to carbohydrates and cancer risk is strongest for the association between fiber and CRC, where increased consumption is associated with reduced risk of disease development and mortality after diagnosis. Results of studies examining complex carbohydrates and risk of cancer types other than CRC generally suggest a protective association, whereas results of studies examining simple carbohydrates and cancer risk are mixed. Future prospective studies and RCTs that include homogenous study populations and consistency in study design and methods of modeling carbohydrate intake are urgently needed. Lastly, given the rapidly growing population of adults living with a history of cancer worldwide, more research should be conducted to determine how varying amount and type of carbohydrates may impact outcomes after cancer diagnosis, including recurrence, survival, second primary cancers, and health-related quality of life [86].
